# Fabrication of Core-Shell Magnetic Molecularly Imprinted Nanospheres towards Hypericin via Click Polymerization

**DOI:** 10.3390/polym11020313

**Published:** 2019-02-13

**Authors:** Xinxin Wang, Yuxin Pei, Yong Hou, Zhichao Pei

**Affiliations:** Shaanxi Key Laboratory of Natural Products & Chemical Biology, College of Chemistry & Pharmacy, Northwest A&F University, Yangling 712100, Shaanxi, China; xinxinwang@nwafu.edu.cn (X.W.); yixilun@126.cn (Y.H.); peizc@nwafu.edu.cn (Z.P.)

**Keywords:** surface molecular imprinting, hypericin, click polymerization, core-shell, magnetic nanospheres

## Abstract

The core-shell structure molecularly imprinted magnetic nanospheres towards hypericin (Fe_3_O_4_@MIPs) were prepared by mercapto-alkyne click polymerization. The shape and size of nanospheres were characterized by dynamic light scattering (DLS) and transmission electron microscope (TEM). The nanospheres were analyzed by FTIR spectroscopy to verify the thiol-yne click reaction in the presence or absence of hypericin. The Brunauer–Emmet–Teller (BET) method was used for measuring the average pore size, pore volume and surface area. The Fe_3_O_4_@MIPs synthesized displayed a good adsorption capacity (*Q* = 6.80 µmol·g^−1^). In addition, so-prepared Fe_3_O_4_@MIPs showed fast mass transfer rates and good reusability. The method established for fabrication of Fe_3_O_4_@MIPs showed excellent reproducibility and has broad potential for the fabrication of other core-shell molecularly imprinted polymers (MIPs).

## 1. Introduction

Molecularly imprinted polymers (MIPs), mimicking a principle similar to antibody–antigen recognition, are synthesized via polymerization to create specific binding sites with memory of the template molecules during the synthetic process of polymers [1]. Molecular imprinting techniques based on MIPs have successfully obtained many applications, including separation [2], solid phase extraction (SPE) [3], chromatography [4], sensors [5], catalysis [6], immunoassay [7], and drug delivery due to the strengths of MIPs [8,9,10], such as high chemical stability, high affinity with templates, and low costs [11].

Commonly, MIPs were prepared by bulk polymerization, precipitation polymerization, and suspension polymerization. Bulky MIPs have the following shortcomings: (1) Inability to completely remove the template; (2) uneven distribution of binding sites; (3) a slow mass transfer rate; (4) and irregular resultant particle size and shape [12,13]. Surface molecularly imprinted polymers (SMIPs) based on core-shell nanoparticles have attracted considerable attention because they create recognition sites on a thin layer, and therefore significantly improve the mass transfer rate. Moreover, elution of templates from SMIPs is easier in comparison with bulky MIPs [14]. Consequently, various hard materials, including silica, graphene, gold–silver nanoparticles, and magnetic materials [15,16], were reported as cores in constructing core-shell MIPs via surface molecularly imprinting approaches. Among them, Fe_3_O_4_ magnetic nanoparticles (MNPs) are often used as cores for their numerous advantages, especially paramagnetic properties. Because the paramagnetic properties of MNPs allow the final core-shell MIPs to be separated quickly and conveniently by a magnet in the separation process, they are highly desired in practical applications [17].

Hypericin (Hyp) has gained increasing interest from researchers due to its bioactivities, such as antidepressant [18], antiviral, antisepsis, antiphlogosis and antitumor properties [19,20,21]. However, extracting Hyp from St John’s wort plant remains a challenge due to the low Hyp concentration of the plant and the shortage of specific adsorbents [22]. Our group has concentrated on fabricating specific adsorbents with high efficiency and high selectivity for Hyp in the past few years. For example, by using Fe_3_O_4_ as the core and polydopamine as the shell, we established a simple way to synthesize the core-shell magnetic molecularly imprinted nanosphere, which possesses great adsorption capacity (*Q* = 18.28 µmol·g^−1^) of Hyp. It also possesses obvious binding selectivity to Hyp [23]. However, the elution of Hyp was difficult due to the strong adsorption of Hyp on polydopamine (PDA).

Since the first report in 2001 [24], click reaction has been broadly applied in many fields such as polymer chemistry, functional materials, surface modification and biochemical sensors because of its high efficiency under mild conditions and fast kinetics. In contrast, only a few works have been published where fabrications of MIPs have been achieved through click reaction [25,26,27,28]. Previously, in order to obtain the polymeric nanospheres, we established a one-step method by using the click reaction between azide and alkyne with ultrasonic assistance in the absence of surfactants [29,30]. The size of the nanospheres could be controlled by using a different ratio of the rational designed monomers to crosslinkers and reaction conditions. Based on this work, we synthesized bulky MIP nanospheres by using tris (3-mercaptopropionate), 3,5-diethynyl-pyridine and Hyp (corresponding to crosslinker, monomer, and template, respectively) by mercapto-alkynyl click polymerization. The polymer nanospheres showed good capacity for adsorption and selectivity toward Hyp (6.80 µmol·g^−1^) [31]. Nevertheless, the separation of MIP nanospheres from the supernatant is difficult and needs a long duration of centrifugation on high speed, which limits their further practical application [32].

**Scheme 1:**
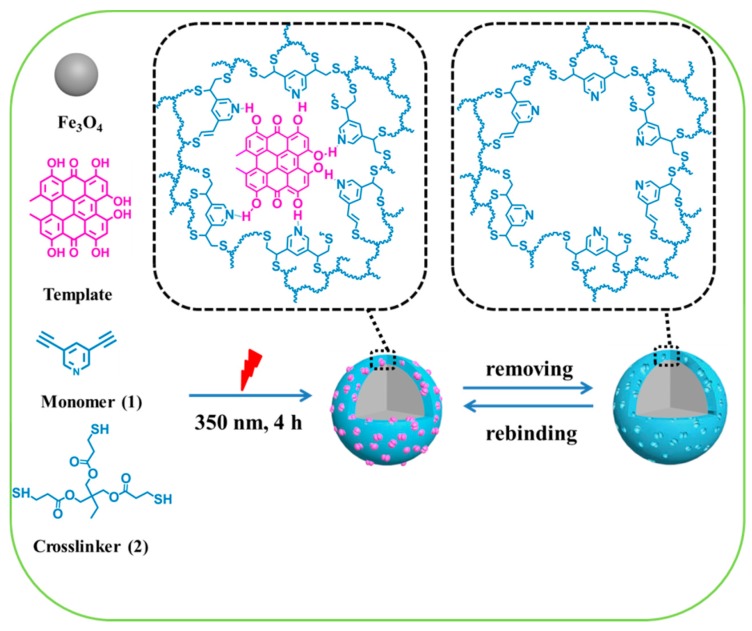
Illustration for the preparation of core-shell structure molecularly imprinted magnetic nanospheres (Fe_3_O_4_@MIPs).

Based on our previous works [23,31], we envision that core-shell magnetic MIP nanospheres (denoted as Fe_3_O_4_@MIPs) can be fabricated by click polymerization between (**1**) 3,5-diethynyl-pyridine, and (**2**) tris (3-mercaptopropionate), on the surface of MNPs in the presence of Hyp. Fe_3_O_4_@MIPs synthesized by this method should facilitate elution of the templates and separation of the nanoshperes from the supernatant. Thus, Fe_3_O_4_@MIPs were synthesized as depicted in Scheme 1 and evaluated accordingly in this work.

## 2. Materials and Methods 

### 2.1. Materials

2-methyl-3-butyn-2-ol was obtained from J&K Scientific Ltd. Benzoin dimethyl ether (DMPA, 98%) was obtained from Tianjin Heowns Biochemical Technology Co., Ltd. (Tianjin, China). 3,5-dibromopyridine (98%), trimethylolpropane (>98%), copper (I) iodide (98%), sodium methylate (97%) and bis (triphenylphosphine) palladium (II) chloride (Pd 15.2%) were obtained from Aladdin (Shanghai, China). Emodin (Emo) was obtained from Tianfeng Biological Technology Co, Ltd. (Xi’an, China). Template molecules were synthesized on the basis of our previous work [33], and characterized by proton nuclear magnetic resonance (^1^H-NMR). The other solvents and chemicals were analytical reagent (AR) grade and used as received without any further purification.

^1^H-NMR spectra were recorded on a Bruker AVANCEIII 500 MHz Spectrometer (Bruker, Fällanden, Switzerland). Transmission electron microscopy (TEM) was performed on an H-600 instrument by Hitachi Ltd. operating at 80 kV. Dynamic light scattering (DLS) (Beckman Coulter, Brea, CA, USA) was used to measure the hydrodynamic diameter of the particles. Nitrogen physisorption (Autosorb-iQ, Quantachrome, Boynton Beach, FL, USA) was used to measure the surface area and the porosity of the nanospheres. High performance liquid chromatography (HPLC): C18 reversed-phase column (5 μm, 4.6 mm × 150 mm, Shimadzu, Kyoto, Japan).

### 2.2. Synthesis of Monomers and Crosslinkers

**Scheme 2:**
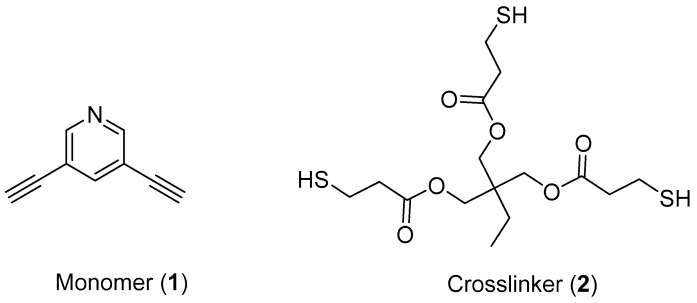
The chemical structures of the monomer (**1**) and the crosslinker (**2**).

In this work, we used compounds **1** and **2** as monomers and crosslinkers for click polymerization (see Scheme 2). Compounds **1** and **2** were synthesized according to the published procedure [29,34] and characterized by ^1^H-NMR (Bruker AVANCE III 500 MHz Spectrometer). The synthetic details can be found in Supplementary Materials and their ^1^H-NMR spectra were shown in Figures S1, S2 and S6–S9.

### 2.3. Preparation of Fe_3_O_4_ MNPs

The Fe_3_O_4_ MNPs were prepared according to the published procedure [35]. FeCl_3_∙6H_2_O (1.35 g, 5 mmol) was added to a beaker with 40 mL ethylene glycol, 1.0 g polyethylene glycol and 3.6 g NaAc. After being vigorously stirred for 30 min, the mixture was sealed in a stainless steel autoclave and kept at 200 °C for 10 h. Then, the mixture was cooled to room temperature to obtain MNPs. The MNPs were washed and stored in absolute ethanol. 

### 2.4. Preparation of Core-Shell Molecularly Imprinted Polymer Magnetic Nanospheres towards Hyp (Fe_3_O_4_@MIPs) via Click Reaction

The mixture containing MNPs (0.216 mmol), 0.1 mmol monomer **1**, 0.1 mmol crosslinker **2**, 0.031 mmol DMPA, 1.5 mL acetone, and 0.5 mL acetonitrile were placed into a quartz tube. The mixture was subjected to ultrasonication for 10 minutes before the addition of a 10 μmol template of Hyp. The final mixture was subjected to UV irradiation (350 nm) for click polymerization on the surface of MNPs for 4 h at an ambient temperature under an inert atmosphere and mechanical stirring to yield Fe_3_O_4_@MIPs.

**Figure 1:**
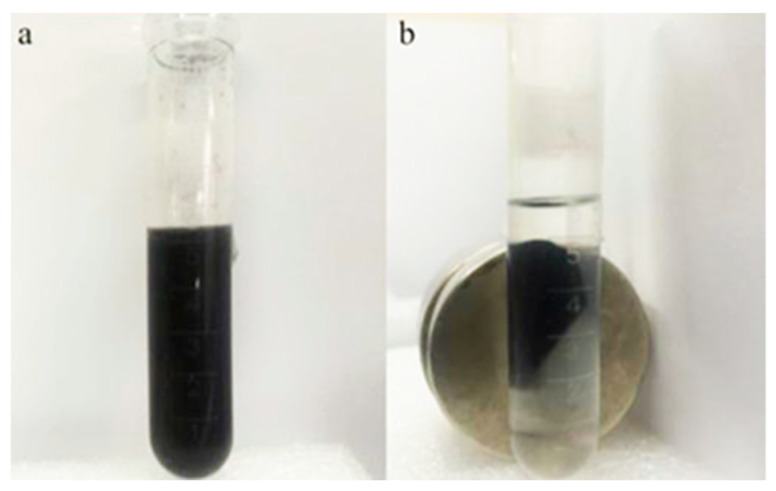
The photos of a suspension of Fe_3_O_4_@MIPs (**a**) and separation by magnets (**b**).

As shown in Figure 1, the final Fe_3_O_4_@MIPs were separated easily from the reaction system with a magnet, due to the paramagnetic property of MNPs, and extracted with acetone containing 10% acetic acid for 48 h in a Soxhlet apparatus, then subjected to ultrasonication in acetone containing 20% acetic acid for 20 min. The ultrasonication step was repeated until no Hyp was detected in the solvent.

The same method was used for preparing the control polymer nanospheres (Fe_3_O_4_@NIPs) in the absence of the template molecules.

### 2.5. Determination of Static Adsorption Capacity

To obtain static adsorption capacity, a test molecule solution in acetone (5 mL, 1.0 μM) and 5 mg Fe_3_O_4_@MIPs (or Fe_3_O_4_@NIPs) were placed into 10 mL centrifuge tubes at 25 °C for 24 h, respectively. HPLC (C18 reversed-phase column, 5 μm, 4.6 mm × 150 mm) was used to measure the free test molecules in the supernatant (Figure S3). The adsorption capacity (*Q*, µmol·g^−1^) was calculated using Equation (1):

(1) $$Q=\frac{(C_{0}-C_{e})\;V}{W}$$

where *C_e_* (μM) and *C*_0_ (μM) are the equilibrium and the initial concentration; *V* (L) is the volume of the solution; and *W* (g) is the dry weight of the nanospheres.

The specific adsorption capacity (*Q*_s_) of Fe_3_O_4_@MIPs is defined as Equation (2):

*Q*_s_ = *Q*_1_ − *Q*_2_ (2)

where *Q*_1_ (µmol·g^−1^) and *Q*_2_ (µmol·g^−1^) are the static adsorption capacity of Fe_3_O_4_@MIPs and Fe_3_O_4_@NIPs, respectively. 

### 2.6. Dynamic Adsorption Test

Fe_3_O_4_@MIPs or Fe_3_O_4_@NIPs (5.0 mg) were added into a centrifuge tube (10 mL). Hyp acetone solution (12.5 μM, 5 mL) was then placed into the tube. There after the tube was wrapped with aluminum foil and shaken in an air bath shaker at 25 °C for different lengths of time (0.5, 1, 2, 4, 8, and 12 h). HPLC was used to measure the concentrations of free Hyp in the supernatants, and each adsorption was calculated using Equation (1). 

### 2.7. Isotherm Adsorption

The nanospheres (5.0 mg) were weighted into six centrifuge tubes with the Hyp acetone solution (5 mL). The concentrations of Hyp acetone solution were 2.5, 5.0, 10.0, 12.5, 25, and 50.0 µM, respectively. Subsequently, the mixtures were shaken at 25 °C for 8 h. HPLC was used to measure the concentrations of Hyp in the supernatants. Each adsorption was then calculated using Equation (1).

### 2.8. Selectivity of Fe_3_O_4_@MIPs and Fe_3_O_4_@NIPs for Hyp

The binding selectivity of the nanospheres was studied according to the following method [23]. Briefly, Hyp, Protohyp, and Emo acetone solution (5 mL, 12.5 μM) were incubated with 5 mg Fe_3_O_4_@MIPs or Fe_3_O_4_@NIPs at 25 °C for 24 h, respectively. The amounts of test molecules bound to the nanospheres were measured by HPLC. The “selectivity factor” (SF) and “imprinting factor” (IF) were used to compare the binding selectivity of the nanoshperes [32], which can be defined by Equations (3) and (4), respectively:

(3) $$D=\frac{C_{0}-C_{e}}{C_{e}}\;SF=\frac{D_{MIP}^{h}}{D_{MIP}^{\prime }}=\frac{(C_{0,\;MIP}^{h}-C_{e,\;MIP}^{h})/C_{e,\;MIP}^{h}}{(C_{0,\;MIP}^{\prime }-C_{e,\;MIP}^{\prime })/C_{e,\;MIP}^{\prime }}$$

where the distribution of hypericin for MIP (L·g^−1^) is represented as $D_{MIP}^{h}$, the distribution of competitor for MIP (L·g^−1^) is represented as *D′_MIP_*, *C*_0_ (µM) is the initial concentration of hypericin, and *C_e_* (µM) is the concentration of hypericin after imprinted.

(4) $$IF=\frac{D_{MIP}}{D_{NIP}}=\frac{(C_{0,\;MIP}-C_{e,\;MIP})/C_{e,\;MIP}}{(C_{0,\;NIP}-C_{e,\;NIP})/C_{e,\;NIP}}$$

where the distribution of the test molecule for MIP (L·g^−1^) is represented as *D_MIP_*, the distribution of the test molecule for NIP (L·g^−1^) is represented as *D_NIP_*, *C*_0_ (µM) is the initial concentration of hypericin, and *C_e_* (µM) is the concentration of hypericin after imprinted.

### 2.9. The Reusability of Fe_3_O_4_@MIPs

Reusability was measured by following the previous report [23]. Fe_3_O_4_@MIPs (5 mg) and Hyp of acetone solution (5 mL, 12.5 μM) were mixed at 25 °C and the mixtures were shaken for 8 h. Following this, HPLC was used for measuring the concentration of Hyp in the supernatant. The adsorption capacity was calculated by Equation (1).

### 2.10. Brunauer–Emmet–Teller Analysis

The average pore size, pore volume and surface area of the nanospheres were analyzed by Autosorb-iQ nitrogen physisorption made by Quantachrome, based on the Brunauer–Emmet–Teller (BET) method. Prior to analysis, the samples were placed into vacuum drying oven at 50 °C for 9 h.

### 2.11. HPLC Analysis

The solution of herb extract was prepared by following a previously reported method [23]. The herb extract solution (8 mL) was mixed with Hyp acetone solution (1 mL, 125 μM) and Protohyp acetone solution (1 mL, 125 μM) to obtain an original solution. 5 mL of this solution was mixed with 5 mg of the nanospheres (Fe_3_O_4_@MIPs or Fe_3_O_4_@NIPs) and shaken for 8 h. The concentration of each compound in the supernatant was measured by HPLC [31]. 

## 3. Results and Discussion

### 3.1. Preparation of Fe_3_O_4_@MIPs

Fe_3_O_4_@MIPs were synthesized according to the procedure described in Scheme 1. MNPs, compound **1**, and compound **2**, were mixed and dealt with ultrasound for 10 min before the template was added. The final mixture was then irradiated by a UV light of 350 nm to initiate the click polymerization on the surface of MNPs. The imprinting sites were created due to the formation of the complex between the template and monomer via π–π interaction, hydrogen bonds and sequential click polymerization. For obtaining the Fe_3_O_4_@MIPs, Hyp molecules were removed.

### 3.2. Characterization of Fe_3_O_4_@MIPs

#### 3.2.1. FTIR Analysis

**Figure 2:**
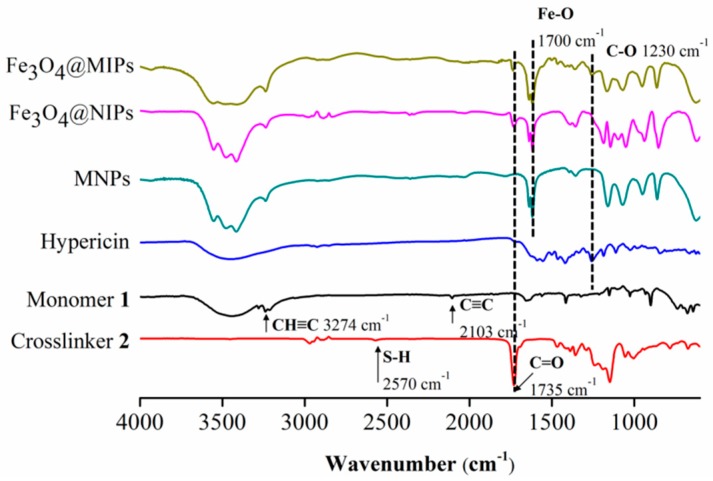
FTIR spectra of Fe_3_O_4_@MIPs, Fe_3_O_4_@NIPs, MNPs, Hyp, monomer **1**, and crosslinker **2**.

The shell of magnetic nanospheres was analyzed by FTIR spectroscopy to verify the thiol-yne click reaction in the presence or absence of Hyp. Their IR spectra, along with those of monomer **1**, crosslinker **2**, Hyp, and MNPs, are shown in Figure 2. It can be seen that, neither characteristic absorptions at 2103 and 3274 cm^−1^ (C≡C and C–H in alkynyl groups) on monomer **1** [29], nor characteristic absorptions at 1735 and 2570 cm^−1^ (C=O and S–H) on crosslinker **2** were observed on both spectra of Fe_3_O_4_@MIPs and Fe_3_O_4_@NIPs. In addition, the absorption band of C–O on Hyp at 1230 cm^−1^ [23] was found on the spectrum of Fe_3_O_4_@MIPs, but not on that of Fe_3_O_4_@NIPs. All these indicated that the thiol-yne click reaction successfully occurred in the presence and absence of Hyp.

#### 3.2.2. Morphological Features

**Figure 3:**
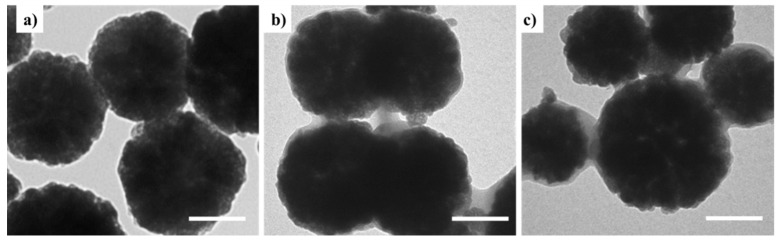
TEM images of MNPs (**a**), Fe_3_O_4_@MIPs (**b**), and Fe_3_O_4_@NIPs (**c**). Scale bar: 100 nm.

Size and morphology of MNPs, Fe_3_O_4_@NIPs and Fe_3_O_4_@MIPs were characterized by H-600 TEM (Hitachi Ltd.) and DLS (Beckman Coulter). Their TEM images are displayed in Figure 3. It can be seen that the original spherical shape of MNPs were kept in the resulting Fe_3_O_4_@MIPs and Fe_3_O_4_@NIPs. Compared to MNPs, a light gray layer around MNPs was observed in both scenarios, which indicated the shell was formed on the surface of the MNPs via click polymerization. 

DLS analysis (Figure S4 and Table 1) showed that the average hydrodynamic diameters of Fe_3_O_4_@MIPs and Fe_3_O_4_@NIPs are 344 and 320 nm, respectively, which is an increase of 35 and 11 nm, respectively, in comparison with that of MNPs (309 nm). The increase of the diameters was due to the formation of a polymer film on the surface of MNPs. The thicker polymer layer of Fe_3_O_4_@MIPs was attributed to the presence of Hyp during polymerization which resulted in the formation of a porous polymer film. The polydispersity indexes of Fe_3_O_4_@MIPs and Fe_3_O_4_@NIPs were 0.386 and 0.315, respectively. Notably, the ζ potential of Fe_3_O_4_@MIPs was changed from 3.49 to −3.58 mV during the extraction process; while the ζ potential of Fe_3_O_4_@NIPs was not affected significantly. The results were consistent with those previously reported [23,31]. This was due to the removal of Hyp molecules from the Fe_3_O_4_@MIPs nanospheres. The result confirms that the Fe_3_O_4_@MIPs can be established by click polymerization between **1** and **2**.

#### 3.2.3. BET Analysis

The BET method was used to measure the average pore size, pore volume and surface area of Fe_3_O_4_@MIPs and Fe_3_O_4_@NIPs (Table 2, Figure S5). The average pore size of Fe_3_O_4_@MIPs is 19.18 nm, nearly 2.5 times higher than that of the Fe_3_O_4_@NIPs, and the surface area and pore volume of Fe_3_O_4_@MIPs are 8.31 m^2^·g^−1^ and 0.04 cm^3^·g^−1^, respectively; which is nearly three times and 10 times higher than that of Fe_3_O_4_@NIPs, respectively. This indicates that the imprinted polymer layer has a porous structure; in contrast, the non-imprinted polymer layer is relatively dense.

### 3.3. Dynamic Adsorption Study

**Figure 4:**
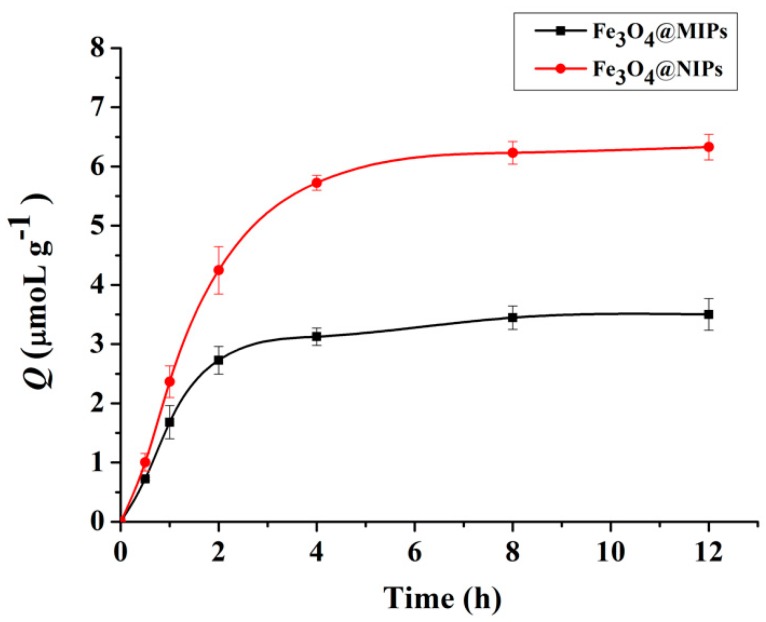
Dynamic adsorption curves of Hyp on Fe_3_O_4_@MIPs and Fe_3_O_4_@NIPs (*n* = 3).

Dynamic adsorption of Fe_3_O_4_@MIPs and Fe_3_O_4_@NIPs were studied. As shown in Figure 4, adsorption amount increased rapidly with time, and reached about 90% at 4 h, which indicates that the Fe_3_O_4_@MIPs obtained good mass transfer properties and that the template molecules reached surface recognition sites relatively easily. Following on from this period, the prolongation of incubation time did not lead to an obvious increase of adsorption. For instance, it took another 4 h to reach the remaining 10% of the total adsorption capacity (equilibrium). While the adsorption amount of Fe_3_O_4_@NIPs towards Hyp under the same conditions, driven by non-specific interaction between the template and the polymer surface, was lower than that of Fe_3_O_4_@MIPs. This was due to the absence of specific recognition sites, small pore volume and low surface area of the Fe_3_O_4_@NIPs.

### 3.4. Affinity Analysis

**Figure 5:**
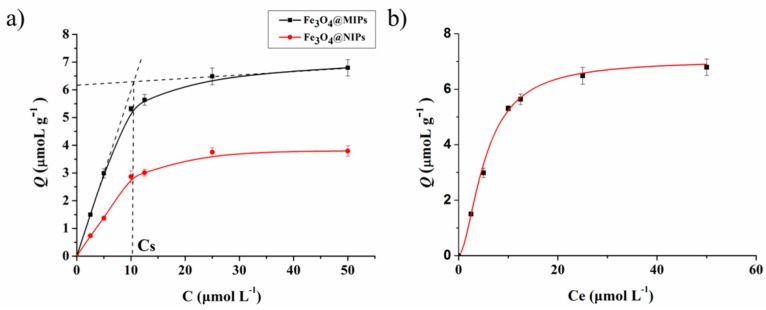
(**a**) The isothermal adsorption curve of Fe_3_O_4_@MIPs and Fe_3_O_4_@NIPs; (**b**) the fitting curve of the extended Langmuir adsorption (*n* = 3).

The ability of Fe_3_O_4_@MIPs for Hyp specific recognition was studied by way of an isothermal adsorption experiment with different concentrations of Hyp (0–50.0 μM). The *Q* values of Fe_3_O_4_@MIPs and Fe_3_O_4_@NIPs are shown in Figure 5a. It can be seen that the binding amount of = Fe_3_O_4_@MIPs increased rapidly in a linear relationship with the concentration of Hyp when it was lower than *C_s_* (11.91 μM). When the concentration reached *C_s_*, the adsorption of Fe_3_O_4_@MIPs reached equilibrium (*Q_max_*) at 6.33·μmol·g^−1^, which is much higher than that obtained with Fe_3_O_4_@NIPs under the same conditions, and can be ascribed to the imprinting effect [22]. Notably, the value of *C_s_* is very close to the one reported previously for bulk MIP nanospheres fabricated via the same click polymerization [31]. This indicates that the property of the polymer film on the surface of so-prepared core-shell Fe_3_O_4_@MIPs is well kept.

The adsorption mechanism was studied by plotting the equilibrium concentrations of Hyp against the corresponding amounts of Hyp bound to Fe_3_O_4_@MIPs. As shown in Figure 5b, it fitted well with the extended model of Langmuir adsorption isotherm expressed by Equation (5) [22,36], where *R*^2^ is 0.9964, *K_d_* = 0.0552 μM, *m* is 1.7052, and *Q_max_* is 7.0524 μmoL·g^−1^. The result proved that the adsorption was monolayer adsorption on a non-smooth surface.

(5) $$\frac{C_{e}^{m}}{Q}=\frac{1}{K_{d}Q_{max}}+\frac{C_{e}^{m}}{Q_{max}}$$

where *C_e_* (μmol·L^−1^) is equilibrium concentration of Hyp in the supernatant, *K_d_* (μM) is the equilibrium constant, and *Q_max_* (μmol·g^−1^) is the maximum adsorption capacity of binding sites.

### 3.5. Binding Selectivity of Fe_3_O_4_@MIPs to the Template Molecule Hyp

**Figure 6:**
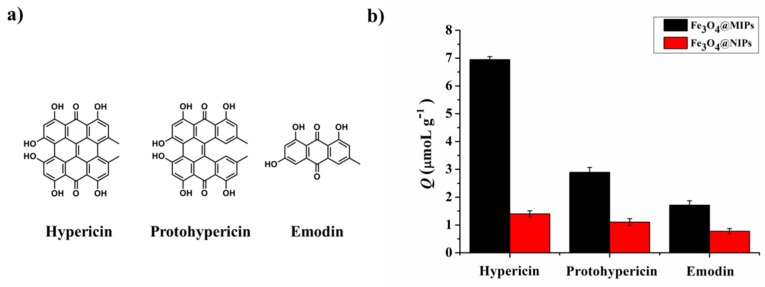
Competitive experiment of Hyp and Hyp structure analogues (*n* = 3).

The binding selectivity of Fe_3_O_4_@MIPs to the template molecule Hyp was measured by a similar method, described in our previous work [23]. Briefly, two phenolic compounds containing phenolic hydroxyl groups, Protohyp and Emo (with chemical structures shown in Figure 6a), were selected as the competitors of Hyp. The respective adsorption capacities of Fe_3_O_4_@MIPs and Fe_3_O_4_@NIPs towards Hyp, Protohyp, and Emo were measured under the same conditions. The results are shown in Figure 6b. The adsorption of Fe_3_O_4_@MIPs towards Hyp was clearly much higher than Protohyp and Emo. In contrast, the respective adsorptions of Fe_3_O_4_@NIPs toward the three different molecules remained similar. 

SF and IF were used to evaluate the binding selectivity of the nanospheres (Table 3). The SF of Fe_3_O_4_@MIPs for Protohyp and Emo were 4.16 and 7.88, respectively. The IF of Fe_3_O_4_@MIPs towards Hyp, Protohyp, and Emo was 9.93, 3.11, and 2.41, respectively. 

### 3.6. Reproducibility and Reusability of Fe_3_O_4_@MIPs

**Figure 7:**
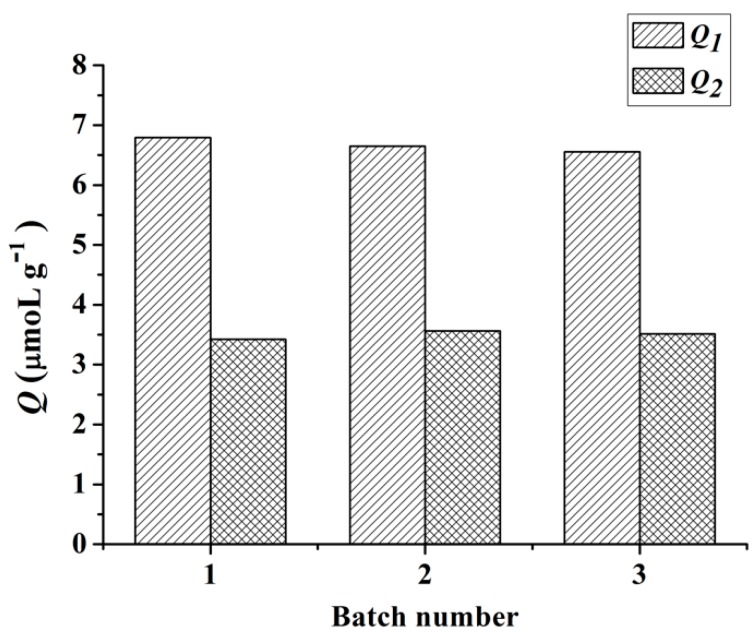
Reproducibility of the method for preparing Fe_3_O_4_@MIPs.

To test the reproducibility of the method in preparing Fe_3_O_4_@MIPs, three parallel experiments were conducted, the products were subjected to adsorption capacity evaluation under the same conditions. As shown in Figure 7, similar adsorption capacities for each individually prepared Fe_3_O_4_@MIPs were obtained (6.80, 6.65, and 6.56 μmol·g^−1^, respectively). The results showed that the click polymerization method used to fabricate Fe_3_O_4_@MIPs was reproducible and that nanospheres can be used for selective identification of Hyp.

**Figure 8:**
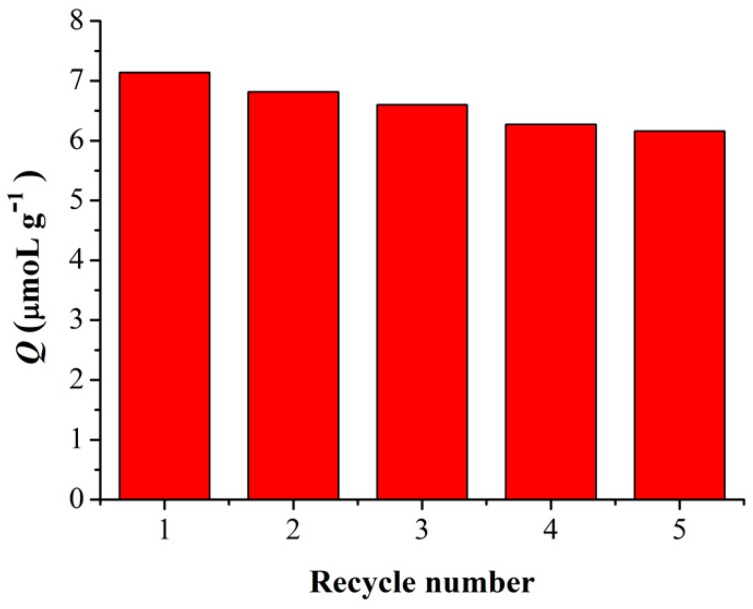
Reusability of Fe_3_O_4_@MIPs.

As a man-made antibody, one of the numerous advantages of MIPs in comparison with native antibodies, is the robustness of the materials which allows for repeated use [37]. Therefore, the reusability of Fe_3_O_4_@MIPs was evaluated and the results are shown in Figure 8. As expected, we found that the adsorption capacity of Fe_3_O_4_@MIPs, similar to its bulky counterpart [31], changed little even after five adsorption–extraction cycles, indicating that nanospheres of Fe_3_O_4_@MIPs have good stability. 

### 3.7. Adsorption of Fe_3_O_4_@MIPs toward Hyp from the Herb Extract 

**Figure 9:**
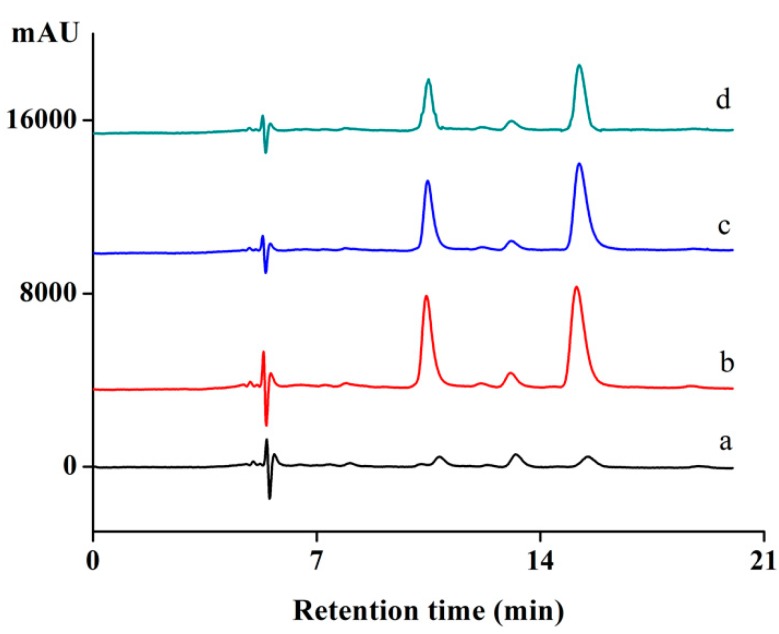
Chromatograms of (**a**) herb extract; (**b**) mixture of extract, Hyp and Protohyp (before adsorption); (**c**) supernatant of Fe_3_O_4_@NIPs (after adsorption); (**d**) supernatant of Fe_3_O_4_@MIPs (after adsorption).

Adsorption of Fe_3_O_4_@MIPs from the herb extract was investigated using a published procedure [23]. To measure selective adsorption ability of Fe_3_O_4_@MIPs towards Hyp, the herb extract was mixed with Hyp and Protohyp (equal amounts). HPLC was used to measure the concentration of Hyp in the supernatant, and the results are shown in Figure 9 and Table 4. As shown in Figure 9, the peaks of Protohyp and Hyp were found. As can be seen in Table 4, 46.5% of Hyp was adsorbed by Fe_3_O_4_@MIPs, while 18.6% was adsorbed by Fe_3_O_4_@NIPs. Here, Fe_3_O_4_@MIPs showed 2.5 times higher adsorption than Fe_3_O_4_@NIPs toward Hyp. However, there was no obvious difference observed for the adsorption of Fe_3_O_4_@NIPs towards Hyp and Protohyp (18.6% and 20.3%, respectively), nor for the adsorption of Fe_3_O_4_@NIPs and Fe_3_O_4_@MIPs towards non-template molecules of Protohyp (20.3% and 21.5%, respectively), which implied that the adsorption was non-specific.

## 4. Conclusions

In conclusion, Fe_3_O_4_@MIPs with core-shell structures were prepared by mercapto-alkyne click polymerization. The Fe_3_O_4_@MIPs showed good specific adsorption towards Hyp and easy separation properties. In addition, Fe_3_O_4_@MIPs showed fast mass transfer rates, good reusability and had potential application in enriching and separating Hyp from herb extracts. Most importantly, this work opens an avenue for the fabrication of core-shell MIPs using click polymerization. Furthermore, MIPs prepared in this method may find potential applications in enrichment, rapid separation, and purification of Hypericin.

## Figures and Tables

**Table 1:** Dynamic light scattering (DLS) and Zeta data of the nanospheres prepared.

Nanospheres	Paticle Size (nm)	Polydispersity Index	ζ Potential (mV)
MNPs	309 ± 61	0.271	1.35 ± 0.17
Fe_3_O_4_@MIPs	344 ± 55	0.386	3.49 ± 0.72
Fe_3_O_4_@NIPs	320 ± 75	0.315	2.06 ± 0.86
Fe_3_O_4_@MIPs after extracting process	323 ± 80	0.646	−3.58 ± 0.58
Fe_3_O_4_@NIPs after extracting process	314 ± 46	0.378	0.20 ± 0.69

**Table 2:** Pore size, surface area, and pore volume of nanospheres.

Nanospheres	Average Pore Diameter (nm)	Surface Area (m^2^·g^−1^)	Pore Volume (cm^3^·g^−1^)
Fe_3_O_4_@MIPs	19.18 ± 0.26	8.31 ± 0.25	0.04 ± 0.03
Fe_3_O_4_@NIPs	7.84 ± 0.12	2.81 ± 0.01	0.004 ± 0.01

**Table 3:** The values of SF and IF.

Factor	Hyp	Protohyp	Emodin
**SF**	-	4.16	7.88
**IF**	9.93	3.11	2.41

**Table 4:** Adsorption capacity of nanospheres for extracts.

Sample	Peak Area (Hyp)	Peak Area (Protohyp)	Adsorption of Hyp (%)	Adsorption of Protohyp (%)
Initial	141,375	80,769	-	-
Fe_3_O_4_@NPs	115,094	64,398	18.6	20.3
Fe_3_O_4_@MIPs	75,618	63,397	46.5	21.5

## Supplementary Materials

The following are available online at http://www.mdpi.com/2073-4360/11/2/313/s1. Figure S1. The Synthesis of monomer **1**; Figure S2. The Synthesis of crosslinker **2**; Figure S3. (**a**) Standard curve of Hyp in acetone by HPLC. (**b**) Standard curve of Protohyp in acetone by HPLC. (**c**) Standard curve of emodin in acetone by HPLC. HPLC detection conditions: C18 reversed-phase column (5 μm, 4.6 mm × 250 mm, Shimadzu, Japan). The mobile phase consisted of 50% acetonitrile, 50% of the mixture of ammonium acetate-acetic acid buffer (0.3 M, pH = 6.96) and methanol (1:4, *v*/*v*); detection wavelength: 590 nm; flow rate: 0.4 mL/min; injection volume: 10 μL; Figure S4. DLS histograms of Fe_3_O_4_@MIPs and Fe_3_O_4_@NIPs before and after extracting process; Figure S5. The nitrogen adsorption and desorption isotherms of Fe_3_O_4_@MIPs and Fe_3_O_4_@NIPs; Figure S6. The ^1^H-NMR spectrum of the monomer **1**; Figure S7. The ^1^H-NMR spectrum of crosslinker **2**; Figure S8. The ^1^H-NMR spectrum of Protohyp; Figure S9. The ^1^H-NMR spectrum of Hyp.
