# Supplementary material for: Fabrication of Core-Shell Magnetic Molecularly Imprinted Nanospheres towards Hypericin via Click Polymerization
Source: Polymers (Basel). 2019 Feb 13;11(2):313. doi: 10.3390/polym11020313 (PMC6419411; doi:10.3390/polym11020313)
Supplement: Supplementary file 1 [file polymers-11-00313-s001.pdf]

Supplementary Materials:
Fabrication of Core-Shell Magnetic Molecularly Imprinted Nanospheres towards Hypericin *via* Click Reaction

Xinxin Wang, Yuxin Pei*, Yong Hou, Zhichao Pei
Shaanxi Key Laboratory of Natural Products & Chemical Biology, College of Chemistry & Pharmacy, Northwest A&F University, Yangling, Shaanxi 712100 (PR China)
* Correspondence: peiyx@nwafu.edu.cn; Tel.: +86-29-87091196; Fax: +86-29-87092769

1. The Synthesis of monomer and crosslinker

3,5-Diethynylpyridine[1]

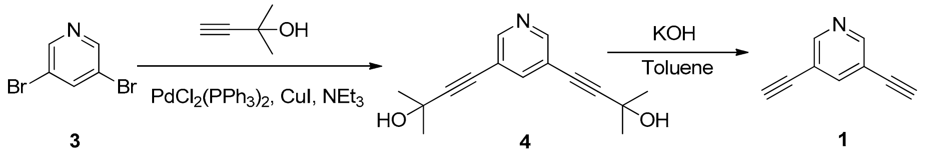

Figure S1. The Synthesis of monomer **1**.

3,5-dibromopyridine (98%) (5.0 g, 21.2 mmol), $PdCl_2(PPh_3)_2$ (450 mg, 0.64 mmol) and CuI (242 mg, 1.27 mmol) were added into 250 mL flask with 50 mL of Ar-degassed triethylamine and 4.5 mL of Ar-degassed 2-methyl-3-butyn-2-ol (98%). The resulting solution was stirred at room temperature for 24 h before the solvent was removed under vacuum. The black residue was extracted with ethyl acetate (15 mL × 5). The organic phase was combined and dried over $MgSO_4$, then filtered through neutral alumina. The filtrate was dried under vacuum to leave black oil. The product was purified by flash column chromatography to give **4** as a yellow powder. (4.75 g, 92%).

Compound **4** (1.0 g, 4.14 mmol) and KOH (0.46 g, 8.25 mmol) were added into 250 mL flask with 50 mL of Ar-degassed toluene. The resulting solvent was refluxed for 12 h. The solvent was removed under vacuum to leave a yellow brown solid, which was then dissolved in dichloromethane. The solution was filtered and the filtrate was dried under vacuum to give a yellow brown solid. The product was purified by flash column chromatography to give **1** as a white powder. (0.258 g, 49%). ^{1}H-NMR (500 MHz, $CDCl_3$) δ 8.65 (d, *J* = 1.9 Hz, 2H), 7.88 (t, *J* = 1.9 Hz, 1H), 3.26 (s, 2H) ppm.

Trimethylolpropane tris(3-mercaptopropionate) [2].

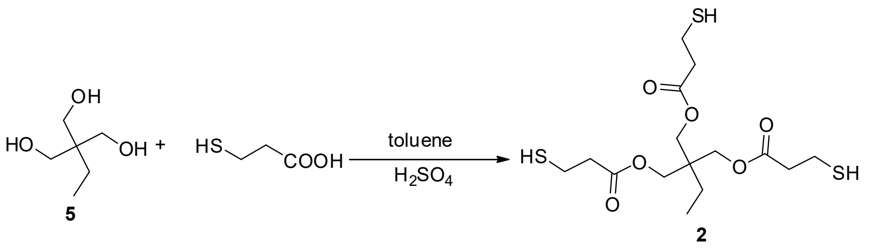

Figure S2. The Synthesis of crosslinker **2**.

1,1,1-tris(hydroxymethyl) propane (3.36 g, 25 mmol), H_2SO_4 (0.201 g, 98%) and toluene (20 mL) were mixed and added in a three-neck flask, which equipped with reflux condensing tube, constant pressure drop funnel, and Dean–Stark trap. 3-mercapto-propanoic acid (8.752 g, 75 mmol) was dropped slowly into the reaction system through the constant pressure drop funnel. And then, the reaction was carried out by heating the flask to 130 °C for 4 h. The reaction was terminated by cooling down to room temperature. Then, the reaction solution was washed with deionized water to neutrality. The organic phase was separated, and dried over anhydrous $MgSO_4$. The transparent oil **2** was obtained by removal of toluene using the rotary evaporator. (8.957 g, 90%). ^{1}H-NMR (500 MHz, $CDCl_3$) δ 0.9 (m, 3H), 1.50 (m, 2H), 1.65 (m, 3H), 2.65 (s, 6H), 2.8 (s, 6H), 4.1 (s, 6H) ppm.

2. The standard curve of Hyp, Protohyp and Emo

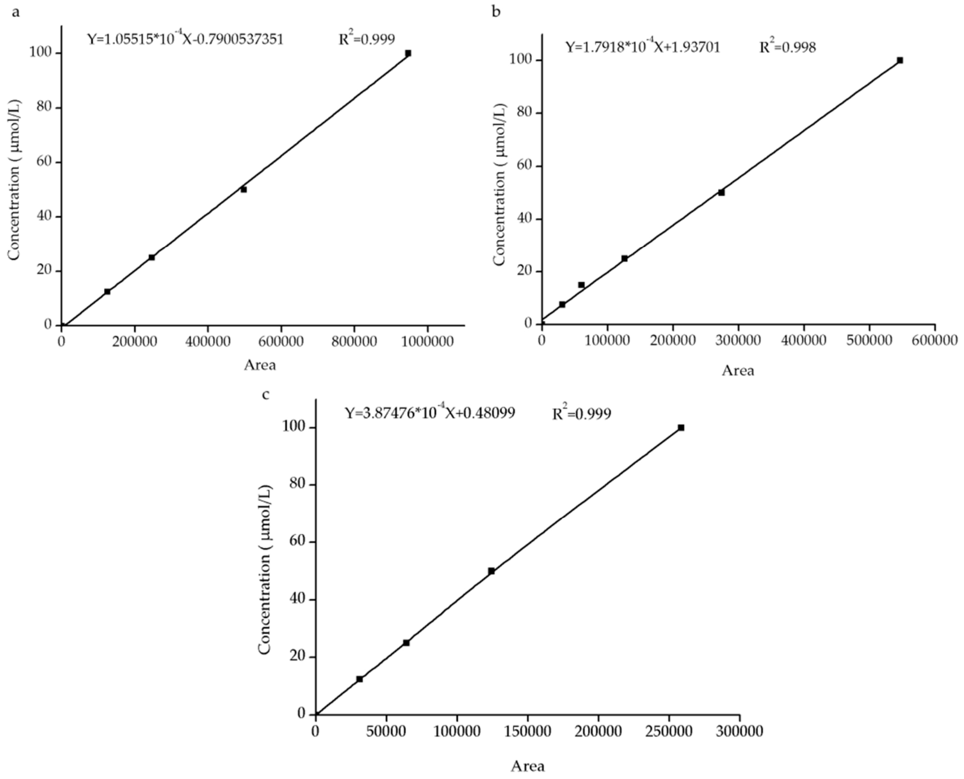

Figure S3. (a) **S**tandard curve of Hyp in acetone by HPLC. (b) Standard curve of Protohyp in acetone by HPLC. (c) Standard curve of emodin in acetone by HPLC. HPLC detection conditions: C18 reversed-phase column (5 μm, 4.6 mm × 250 mm, Shimadzu, Japan). The mobile phase consisted of 50% acetonitrile, 50% of the mixture of ammonium acetate-acetic acid buffer (0.3 M, pH = 6.96) and methanol

(1:4, v/v); detection wavelength: 590 nm; flow rate: 0.4 mL/min; injection volume: 10 μL.

3. DLS Analysis

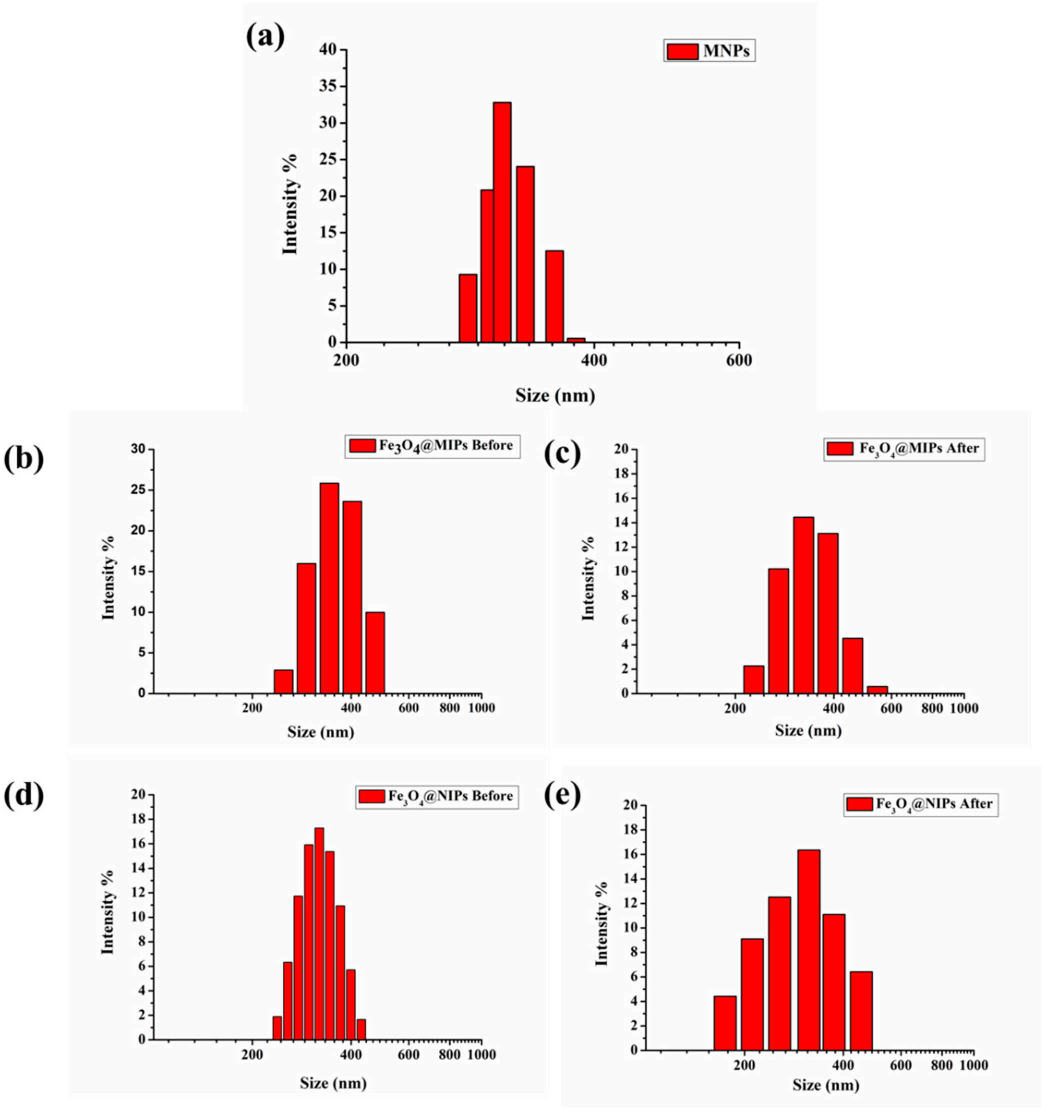

Figure S4. DLS histograms: MNPs (a), Fe_3O_4@MIPs and Fe_3O_4@NIPs before (b, d) and after (c, e) extracting process.

4. BET analysis

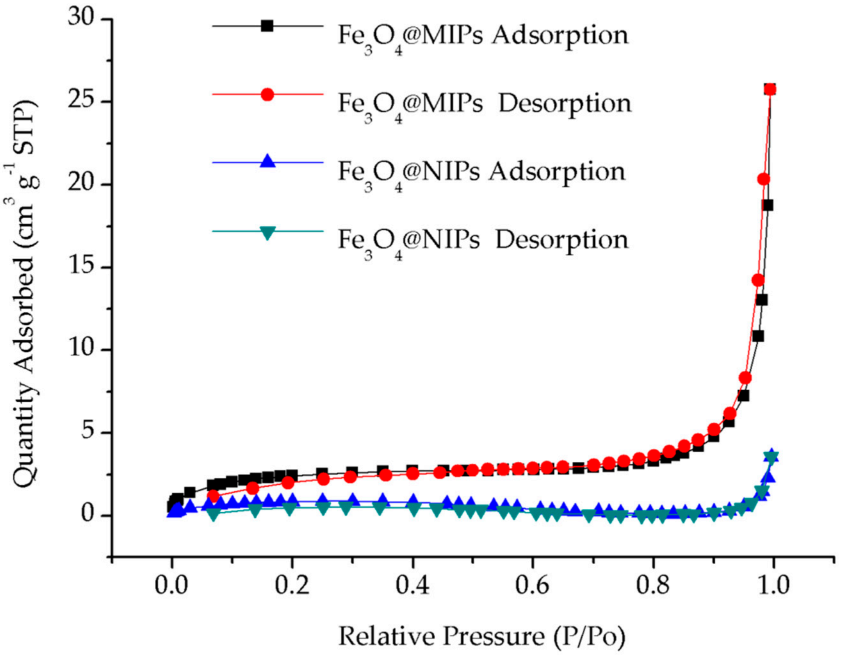

Figure S5. The nitrogen adsorption and desorption isotherms of Fe_3O_4@MIPs and Fe_3O_4@NIPs .

5. The ^{1}H-NMR spectra of compounds

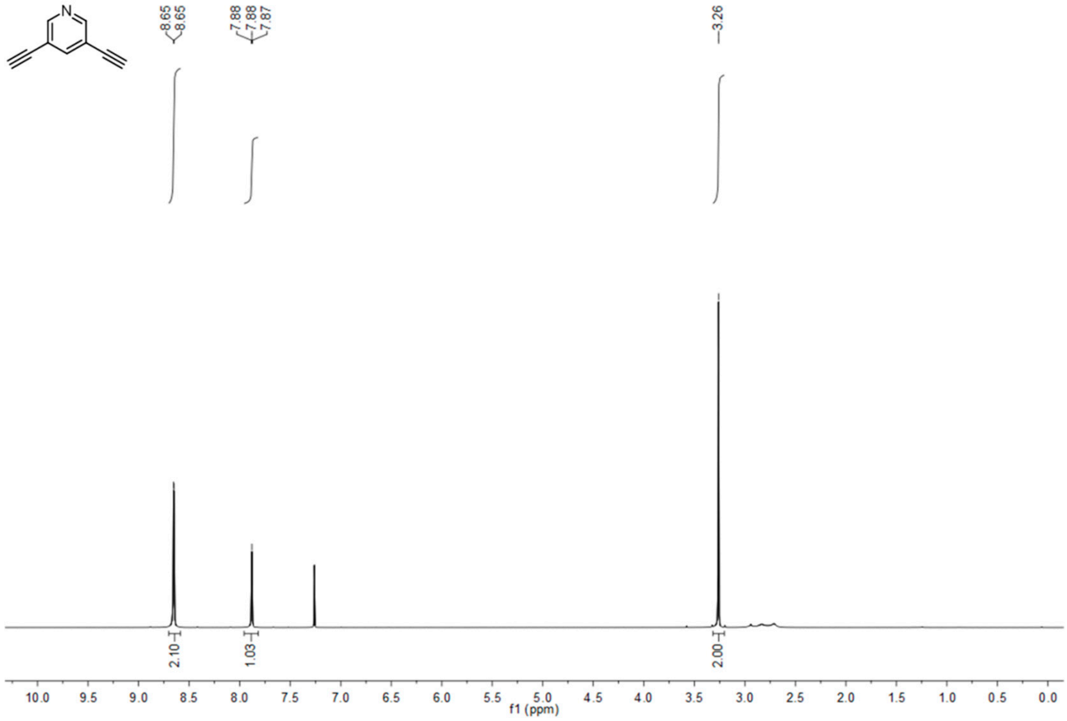

Figure S6. The ^{1}H-NMR spectrum of the monomer **1**.

Monomer 1: ^{1}H-NMR (500 MHz, CDCl$_3$) δ 8.65 (d, J = 1.9 Hz, 2H), 7.88 (t, J = 1.9 Hz, 1H), 3.26 (s, 2H) ppm.

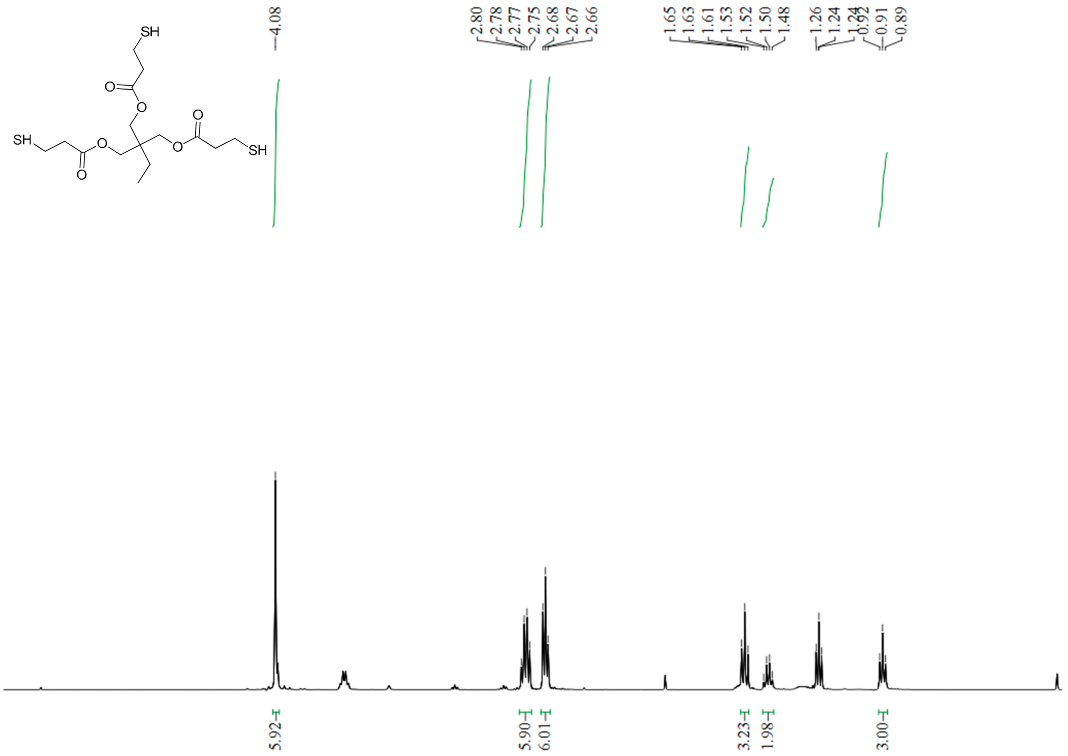

Figure S7. The ^{1}H-NMR spectrum of crosslinker **2**.

Crosslinker 2: ^{1}H-NMR (500 MHz, CDCl$_3$) δ 0.9 (m, 3H), 1.50 (m, 2H), 1.65 (m, 3H), 2.65 (s, 6H), 2.8 (s, 6H), 4.1 (s, 6H) ppm.

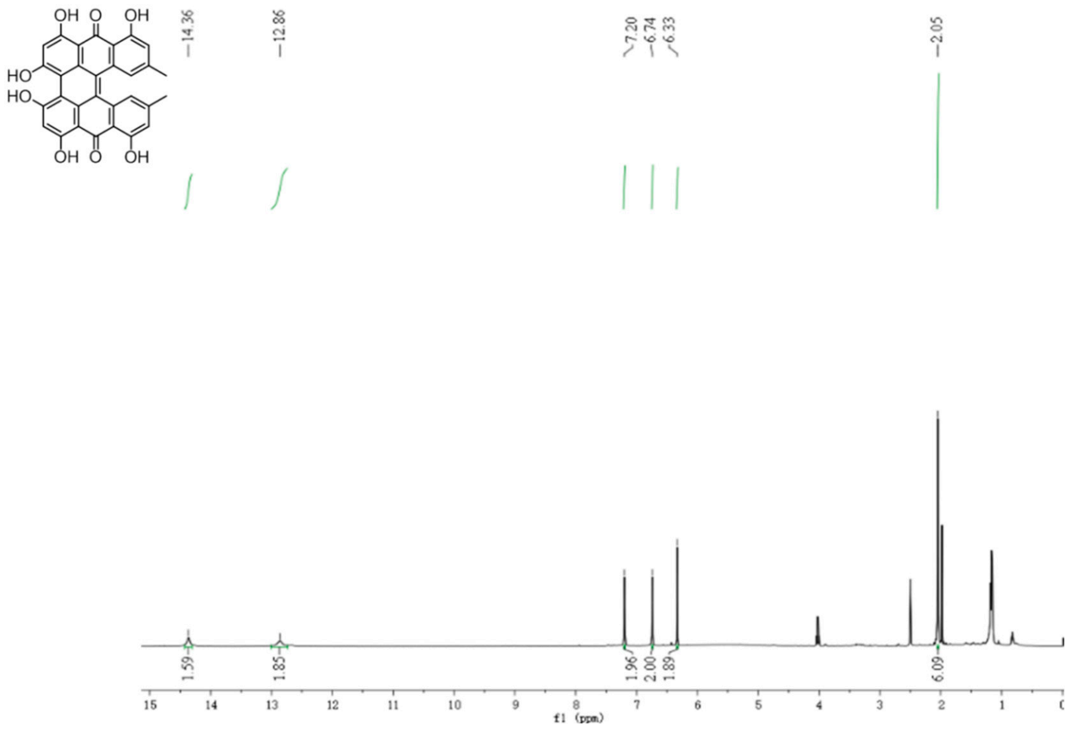

Figure S8. The ¹H-NMR spectrum of Protohyp.

Protohyp: ^{1}H-NMR (500 MHz, DMSO-d_6) δ 14.36 (s, 2H), 12.86 (s, 2H), 7.20 (s, 2H), 6.74 (s, 2H), 6.33 (s, 2H), 2.05 (s, 6H).

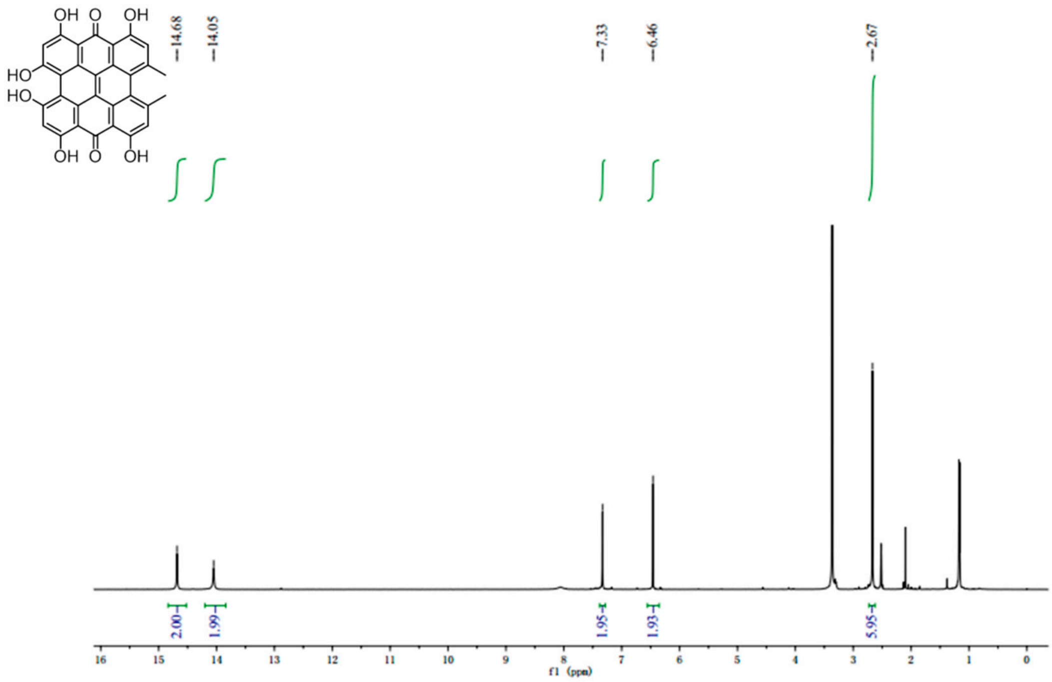

Figure S9. The ^{1}H-NMR spectrum of Hyp.

Hyp: ^{1}H-NMR (500 MHz, DMSO-d_6) δ 14.68 (s, 2H), 14.0 (s, 2H), 7.33 (s, 2H), 6.46 (s, 2H), 2.67 (s, 6H).

References

1. Sun, S.S.; Lees, A.J. Synthesis and photophysical properties of dinuclear organometallic rhenium(i) diimine complexes linked by pyridine-containing macrocyclic: Phenylacetylene ligands. *Organometallics* **2001**, *20*, 2353-2358.
2. Jin, F.; Zheng, M.L.; Zhang, M.L.; Zhao, Z.S.; Duan, X.M. A facile layer-by-layer assembly method for the fabrication of fluorescent polymer/quantum dot nanocomposite thin films. *Rsc Adv* **2014**, *4*, 33206-33214.
